# Dexamethasone Modulates the Cytokine Response but Not COVID-19-Induced Coagulopathy in Critically Ill

**DOI:** 10.3390/ijms24087278

**Published:** 2023-04-14

**Authors:** Mélanie Dechamps, Julien De Poortere, Marie Octave, Audrey Ginion, Valentine Robaux, Laurence Pirotton, Julie Bodart, Damien Gruson, Marie-Astrid Van Dievoet, Jonathan Douxfils, Hélène Haguet, Laure Morimont, Marc Derive, Lucie Jolly, Luc Bertrand, Pierre-François Laterre, Sandrine Horman, Christophe Beauloye

**Affiliations:** 1 Pôle de Recherche Cardiovasculaire (CARD), Institut de Recherche Expérimentale et Clinique (IREC), Université Catholique de Louvain (UCLouvain), 1200 Brussels, Belgium; 2 Department of Cardiovascular Intensive Care, Cliniques Universitaires Saint-Luc, 1200 Brussels, Belgium; 3 Department of Clinical Biology, Cliniques Universitaires Saint-Luc, 1200 Brussels, Belgium; 4 Department of Pharmacy, Namur Research Institute for Life Sciences (Narilis), 5000 Namur, Belgium; 5 Qualiblood, s.a., 5000 Namur, Belgium; 6 Inotrem s.a., 54500 Vandoeuvre-les-Nancy, France; 7 Walloon Excellence in Life Sciences and Biotechnology (WELBIO) Department, WEL Research Institute, 1300 Wavre, Belgium; 8 Department of Intensive Care, Centre Hospitalier Regional Mons-Hainaut, 7000 Mons, Belgium; 9 Critical Care Coordinating Center (4Cs), 1200 Brussels, Belgium; 10 Department of Cardiology, Cliniques Universitaires Saint-Luc, 1200 Brussels, Belgium

**Keywords:** COVID-19, low dose dexamethasone, inflammation, coagulopathy, cytokine storm

## Abstract

Severe forms of coronavirus 2019 (COVID-19) disease are caused by an exaggerated systemic inflammatory response and subsequent inflammation-related coagulopathy. Anti-inflammatory treatment with low dose dexamethasone has been shown to reduce mortality in COVID-19 patients requiring oxygen therapy. However, the mechanisms of action of corticosteroids have not been extensively studied in critically ill patients in the context of COVID-19. Plasma biomarkers of inflammatory and immune responses, endothelial and platelet activation, neutrophil extracellular trap formation, and coagulopathy were compared between patients treated or not by systemic dexamethasone for severe forms of COVID-19. Dexamethasone treatment significantly reduced the inflammatory and lymphoid immune response in critical COVID-19 patients but had little effect on the myeloid immune response and no effect on endothelial activation, platelet activation, neutrophil extracellular trap formation, and coagulopathy. The benefits of low dose dexamethasone on outcome in critical COVID-19 can be partially explained by a modulation of the inflammatory response but not by reduction of coagulopathy. Future studies should explore the impact of combining dexamethasone with other immunomodulatory or anticoagulant drugs in severe COVID-19.

## 1. Introduction

Coronavirus disease 2019 (COVID-19), is an airborne viral infection due to Severe Acute Respiratory Syndrome Coronavirus 2 (SARS-CoV-2). SARS-CoV-2 enters organs by binding its spike glycoprotein to the angiotensin-converting enzyme 2 receptor. The local immune response triggers a progressive systemic inflammatory reaction, implicating endothelial cells, monocytes, macrophages, neutrophils, and lymphocytes. Severe forms of COVID-19 are driven by a dysregulated inflammatory reaction, also called “cytokine storm”, characterized by high systemic levels of cytokines, including tumor necrosis factor α (TNFα), interleukine-1 (IL-1), interleukine-6 (IL-6), and interferon γ (IFNγ) [1,2,3,4,5,6,7].

Inflammatory reactions, immune system and endothelial cell activation and dysfunction lead to an activation of the coagulation system in a phenomenon called “immunothrombosis” involving the formation of Neutrophil Extracellular Traps (NETs) [5,8,9,10,11,12,13,14,15] NETs formation is an innate immune response that neutrophils deploy in addition to phagocytosis to constrain the spread of microorganisms. They are made of large networks of extracellular DNA fibers that are assembled with cytosolic and granule proteins such as histones [16]. NETs are known to be pro-coagulant due to their intrinsic capacity to activate platelets and bind coagulation factors [17]. Accordingly, severe forms of COVID-19 can develop a coagulopathy called COVID-19-associated coagulopathy (CAC), leading to deep venous thrombosis, pulmonary embolisms, arterial thrombosis, and an increased mortality [18,19,20]. CAC had been initially assimilated to a disseminated intravascular coagulopathy (DIC) but is currently recognized to be a specific coagulopathy characterized by elevated plasmatic levels of von Willebrand factor (vWF), soluble P-selectin (sP-selectin), tissue factor (TF), tissue factor pathway inhibitor (TFPI), and thrombin–antithrombin complexes (TAT). As opposed to sepsis, lower platelet, fibrinogen and antithrombin consumption, and less fibrinolysis alteration is seen [4,11].

Considering the pathophysiology of critical COVID-19, several anti-inflammatory, immunomodulatory or anticoagulant medications have been explored as a treatment [21]. Therapeutic anticoagulation, compared with standard low- or intermediate-dose thromboprophylaxis, has shown conflicting results, and seems to be beneficial for hospitalized but not critically ill patients [22,23,24,25]. Among anti-inflammatory medications, low-dose dexamethasone (DXM) is the only treatment that was shown to reduce the risk of mortality for the patients with respiratory insufficiency requiring oxygen supplementation [26,27,28,29,30,31].

Steroids are known to bind to the glucocorticoid receptors present in the cytoplasm of almost all cells and travel to the nucleus, directly or indirectly modulating the transcription of many genes implicated in the inflammatory response. The main expected effect is the inhibition of the synthesis of inflammatory mediators such as pro-inflammatory ILs, TNFα, and IFNγ. At the same time, steroids usually promote the biosynthesis of anti-inflammatory cytokines, including IL-10 [32], inhibiting neutrophil adhesion to endothelial cells and macrophage activation [33,34]. In severely ill COVID-19 patients, DXM administration has been shown to reduce plasma levels of several cytokines at day seven after intensive care unit (ICU) admission [35]. However, changes in coagulopathy following steroid administration in severe COVID-19 have not been explored. One meta-analysis found a higher likelihood of COVID-19 patients presenting with venous thromboembolism when administered corticosteroids [36], suggesting the medication could worsen the coagulopathy. Furthermore, the use of steroids has significant potential adverse effects, mainly their immunosuppressive action that could promote secondary bacterial infection in COVID-19 ventilated patients [29,37]. Therefore, it would be of major importance to determine the underlying changes driving DXM effects in severe COVID-19, looking for the opportunity to explore targeted anti-inflammatory, immunomodulatory, or anticoagulant medications as alternatives with less side effects or as adjunct therapies to increase efficacity.

The present study aimed to prospectively compare plasma inflammation and coagulation biomarkers in patients admitted to the ICU for critical forms of COVID-19 treated with or without DXM.

## 2. Results

### 2.1. Patients Baseline Characteristics

Overall, 22 patients were included in the no DXM group and 24 in the DXM group. The mean age was 59.9 ± 10.4 years and 64.5 ± 8.9 years, respectively (*p* = 0.12). There were no significant differences in the main comorbidities between the two groups. The patients included in the DXM group had more severe respiratory failure and, accordingly, a higher sequential organ failure assessment (SOFA) score. None of the patients met the criteria for sepsis-induced coagulopathy (SIC) nor DIC. The delay between symptom onset and inclusion was similar in both groups (10 ± 5 days in the no DXM group and 10 ± 4 in the DXM group). The duration of treatment with DXM before inclusion was 3.0 ± 2.6 days. Throughout the inclusion period, the SARS-CoV-2 variant was the initial Alpha variant.

### 2.2. Clinical Outcome

The mean ventilation duration and ICU length of stay were longer for the patients in the DXM group. The 30-day and 1-year mortality rates were 27% and 36% for the no DXM group and 37% and 50% for the DXM group. The patients’ baseline characteristics and clinical outcome are described in Table 1.

### 2.3. Endothelial Activation and Coagulopathy (Figure 1)

The study showed no difference in the level of plasmatic endothelial and coagulopathy biomarkers between the two groups (intercellular adhesion molecule [ICAM], vascular cell adhesion molecule [VCAM], TF, TFPI, vWF, tissue plasminogen activator [tPA], plasminogen activator inhibitor-1 [PAI-1], thrombin–antithrombin complex [TAT], international normalized ratio [INR], antithrombin, D-Dimers, and Fibrinogen) except for VCAM which was more elevated in the DXM group. INR was slightly prolonged in both groups (1.23 ± 0.10 and 1.22 ± 0.13) and there was no antithrombin consumption (87 ± 11% and 96 ± 21%).

### 2.4. Platelet Activation and NETosis (Figure 2)

On average, the platelet count was in the normal range in both groups (278,000 ± 115,000/μL and 277,000/μL ± 104,000/μL) with similar levels of sP-selectin (50 ± 22 and 43 ± 21 ng/mL) as a biomarker of platelet activation. NETosis was assessed using citrullinated histone 3 (Cit-H3), neutrophil elastase (NE), and myeloperoxidase (MPO). Cit-H3 and NE remained unchanged but MPO was significantly reduced in the DXM group (405.8 ± 343.9 ng/mL and 196.8 ± 111.1 ng/mL).

### 2.5. Inflammatory Reaction and Immune Response

**Figure 3:**
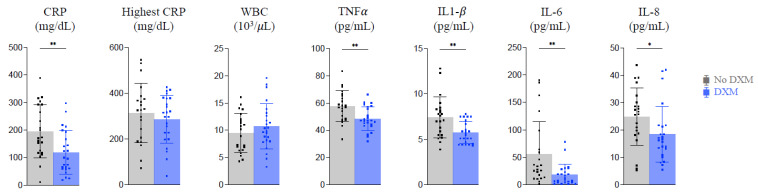
Corticosteroid effects on ubiquitous inflammatory reaction in critical COVID-19 patients. Scatter graphs of ubiquitous proinflammatory cytokines. Individual values (dots), mean (colored rectangle), and standard deviation are presented on the graphs. * *p* < 0.05, ** *p* < 0.01 between the no DXM group and the DXM group. CRP, C-reactive protein; IL, interleukin; TNF α, tumor necrosis factor α; WBCs, white blood cells.

**Figure 4:**
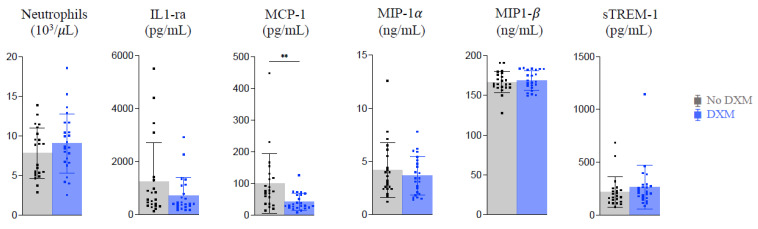
Corticosteroid effects on myeloid immune response in critical COVID-19 patients. Scatter graphs of myeloid inflammatory cytokines. Individual values (dots), mean (colored rectangle), and standard deviation are presented on the graphs. ** *p* < 0.01 between the no DXM group and the DXM group. IL, interleukin; IL-1ra, IL-1 receptor antagonist; MCP-1, monocyte chemoattractant protein 1; MIP, macrophage inflammatory protein-1α; sTREM-1, soluble triggering receptor expressed on myeloid cells 1.

**Figure 5:**
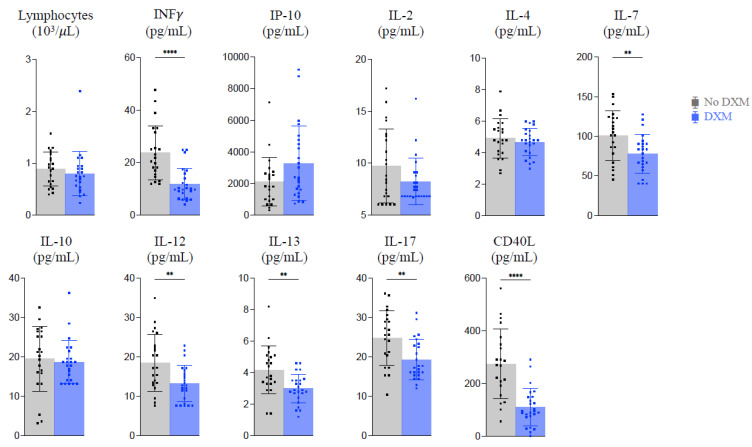
Corticosteroid effects on lymphoid immune response in critical COVID-19 patients. Scatter graphs of lymphoid inflammatory cytokines. Individual values (dots), mean (colored rectangle), and standard deviation are presented on the graphs. ** *p* < 0.01, **** *p* < 0.0001 between the no DXM group and the DXM group. CD40L, CD40 ligand; IFNγ, interferon gamma; IL, interleukin; IP-10, interferon gamma-induced protein 10.

Ubiquitous inflammation biomarkers C-reactive protein (CRP), IL-1β, IL-6, IL-8, and TNFα were all significantly reduced in the DXM group (Figure 3). White blood cell and neutrophil counts at ICU admission were similarly elevated in both groups. Among the plasmatic biomarkers of myeloid immune response activation, IL-1ra, MCP-1, MIP-1α, MIP1-β, and TREM-1, only MCP-1 was significantly reduced in the DXM-treated group (Figure 4). The lymphocyte count was unchanged between the groups. Among the plasmatic biomarkers of lymphoid immune response activation, INFγ, IL-7, IL-12, IL-13, IL-17, and sCD40L were all significantly reduced in the DXM group whereas the levels of IL-2, IL-4, IL-10, and interferon gamma-induced protein 10 (IP10) were similar (Figure 5).

**Figure 6:**
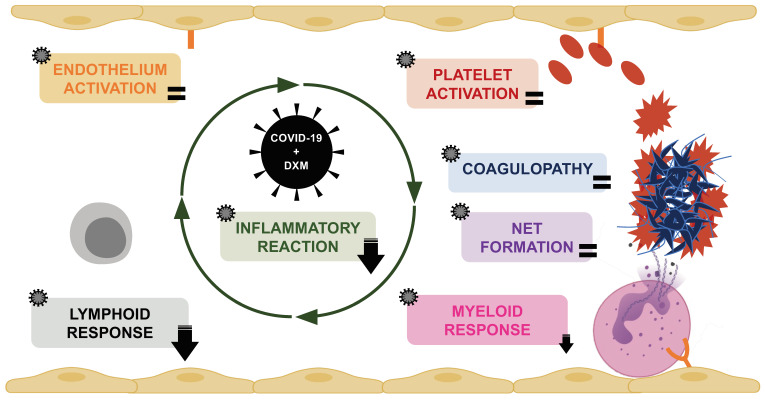
Overview of corticosteroid effects on the inflammatory and immune responses, endothelial and platelet activation, NETosis, and coagulopathy in patients with severe COVID-19.

Figure 6 provides an overview of corticosteroid effects on the inflammatory and immune responses, endothelial and platelet activation, NETosis, and coagulopathy in patients with severe COVID-19.

## 3. Discussion

In this study, low dose DXM systemic administration did not influence CAC in critical COVID-19 patients but, as expected, reduced the plasma levels of circulating pro-inflammatory cytokines [32]. With regards to the immune response, DXM mostly affected the lymphoid immune response but had several impacts on the myeloid immune response. This observation could be explained by the fact that the immune response during severe forms of COVID-19 is characterized by an important lymphoid immune response, similar to SARS-CoV1 infection and as opposed to severe bacterial infections [4,38]. The effects of steroids may, therefore, be more marked on the most important changes in the inflammatory response that are normally caused by the virus. Of note, DXM did not increase the level of the anti-inflammatory interleukins IL1ra and IL10.

However, although the occurrence of the CAC is related to severe inflammatory and immune responses [10,11,12], the reduction of the latter using low dose DXM did not affect the coagulopathy. In line with this, endothelial activation and NETosis formation, which are also closely related to coagulopathy [11], were not affected by DXM treatment. Among plasma biomarkers of endothelial activation, the levels were similar between the two patient cohorts and even higher in corticosteroid-treated patients with respect to VCAM. Endothelial dysfunction in COVID-19 is not only related to the inflammatory response, but also because SARS-CoV-2 directly infects vascular endothelial cells, leading to cell damage and apoptosis and reducing their normal anti-thrombotic activity [39,40]. Regarding NETS formation, MPO was significantly reduced in corticosteroid-treated patients in contrast to the Cit-H3 level, which remained elevated and is a better biomarker of actual NETs formation [41]. The decrease in MPO is, therefore, probably related to the reduction of myeloid activation and not to a reduction in NETs formation. As previously described [4], critical COVID-19 patients had a very high level of circulating TF, unaltered by steroid treatment, and TF is known to be a key player in the initiation of CAC. Hence, the outcome improvement that was observed on DXM for COVID-19 patients requiring oxygen [26,27,28,29,30] is not related to a decrease in the coagulopathy. The present study reinforces data that prove that CAC is a distinct entity from SIC and DIC, without platelet consumption, without AT consumption, only a slight prolongation of the INR, and a high fibrinogen and D-Dimer level. Accordingly, none of the patients met the criteria for SIC nor DIC diagnosis [42,43]. As CAC is associated with a worse prognosis for patients [18], efforts should be made to find a specific treatment for CAC to use in addition to DXM.

Our study is in line with previous descriptions of the role of steroids on sepsis-induced coagulopathy. In healthy volunteers exposed to LPS, prednisolone reduced cytokines release of TNF-alpha and IL-6 but had no impact on LPS-induced coagulation activation. Soluble TF plasma levels were not modified by prednisolone [44]. With regard to COVID-19, two studies showed results that were consistent with our study. In the first, for patients admitted to the general ward with lower severity scores than in our study, DXM that was administered within 19 h of collection reduced plasma IL-6 and IL-1ra levels but did not influence COVID-19 coagulation disorders [45]. In the second, for patients admitted to intensive care, DXM decreased plasma levels of cytokines (IL-1, IL-2, IL-6, IL-8, IL-10, IL-17, IFNγ, and MIP-1) and endothelial markers but biomarkers of coagulopathy were not measured [35]. A third study reported that DXM use in severe COVID-19 patients was associated with a decrease of both proinflammatory and procoagulant profiles, however, the influence of DXM on coagulation disorders was indirectly estimated by a reduction in the need for heparin over time but no data on procoagulant or anticoagulant markers were provided [46].

Of note, hydroxychloroquine (HCQ) was given to the vast majority of patients in the no DXM group. This drug was indeed part of the usual treatment regimen during the first months of the pandemic. HCQ has immunomodulatory actions including inhibition of immune complex-mediated Toll-like receptors 7 and 9 preventing downstream type-1 interferon transcription. HCQ also decreases T-cell- and B-cell-mediated cytokine release [47]. Therefore, it may be argued that plasma cytokine levels could have been modulated by this drug in the no DXM group, decreasing the between-group differences with the DXM group. Yet, there is no published study comparing the level of the inflammatory biomarkers in COVID-19 patients that were treated or not with HCQ. In a cohort of seven patients with mild respiratory symptoms, the impact of HCQ on cytokine kinetics remained unclear [48]. In our cohort, the three patients in the no DXM group who did not receive HCQ had cytokine levels similar to the overall group and did not influence the study results. Moreover, HCQ has no known effect on coagulation disorders.

In this study, patients included in the DXM group had more severe respiratory failure and a higher mortality rate than patients in the no DXM group. Indeed, the present study was not designed to show an impact on clinical outcome and the anti-inflammatory effect of DXM may have been underestimated by the higher severity of patients in the DXM group.

This study has several limitations. Firstly, the number of patients that were recruited was limited and the findings would need to be confirmed in a larger population. Changes in SARS-Cov2 variants, the decrease in COVID-19 severity, and the corresponding decrease in ICU admissions prevented further inclusion of patients in our study. Standard DXM treatment will of course prevent the recruitment of additional patients not treated with DXM. Secondly, all biomarkers were determined in a single sampling within the first days of ICU admission and their time course was not assessed. Finally, plasma biomarkers do not assess cellular reactivity and do not fully reflect the inflammatory and immune response since many effectors are located on the cell surface.

## 4. Materials and Methods

### 4.1. Design and Setting of the Study

This before and after intervention analysis comparing clinical outcomes, inflammatory reaction, and coagulopathy between critical COVID-19 patients that were treated or not with DXM was a sub-study of a monocenter, prospective, translational observational study. Adult patients were systematically included between 30 March 2020 and 11 November 2020.

### 4.2. Population

Patients with critical COVID-19 were those admitted to the ICU for moderate or severe ARDS due to SARS-CoV-2 infection. They were included within five days of admission. ARDS was diagnosed according to the Berlin definition [49], and SARS-CoV-2 infection was demonstrated by real-time reverse transcription PCR on nasopharyngeal swabs. The exclusion criteria were patients on antibiotics for any suspected or confirmed bacterial coinfections, therapeutic anticoagulation (oral or parenteral, including heparins, fondaparinux, vitamin K antagonists, and direct oral anticoagulants), recent (within less than one month) chemotherapy, active inflammatory disease, hemophilia and other coagulopathies, previous history of thrombocytopenia (<100,000 platelets/mm^3^), cirrhosis (Child–Pugh > A), recent (within less than 48 h) major surgery, cardiac arrest during ICU stay, and decision of care limitation. All patients received thromboprophylaxis using low-molecular-weight heparin (LMWH; nadroparin 3800 IU/days subcutaneously) and sampling was performed at least six hours after LMWH injection. Patients included in the first wave of COVID-19 (1 March to 30 June 2020) were compared with patients included in the second wave of COVID-19 (1 October to 30 December 2020), the latter having been routinely treated with low dose DXM on admission to hospital with oxygen supplementation. The treatment consisted of 6 milligrams of DXM per day, administered orally, for a period of 10 days.

### 4.3. Clinical Outcomes

Patient baseline characteristics and clinical outcomes were compared. Patient prognosis was assessed using acute physiologic assessment and chronic health evaluation II (APACHE II) [50] and SOFA [51] scores. Moreover, DIC and SIC were diagnosed using the International Society of Thrombosis and Haemostasis scoring at inclusion [42,43]. Data were collected from central medical records, including biological datasets that were routinely performed in patients that are admitted to the ICU such as platelet count, CRP level, coagulation assessment, renal function, and liver enzymology. Clinical outcomes were assessed 30 days and 1 year after ICU admission.

### 4.4. Sampling

Blood samples were collected through the central venous catheter using vacutainer tubes containing CPDA. After two centrifugation runs enabling platelet isolation, plasma was collected, apportioned into 1 mL aliquots, and stored at −80 °C until use.

### 4.5. Measurement of Biomarkers

Soluble biomarkers of inflammation, coagulation, endothelial and platelet activation, and NETosis were measured using an enzyme-linked immunosorbent assay (ELISA) or suspension array sandwich immunoassays according to regulatory requirements for commercially available research use only. Frozen platelet poor plasma was thawed at room temperature the day of the experiment. The details of each marker that was analyzed are listed in the Supplemental Table S1. Each analytical run was performed in duplicate. The methods respected their respective validated lower limit of quantification and upper limit of quantification. Cytokines and chemokines were measured using Bio-Plex Pro Human Cytokine 27-Plex Panel (27-Plex) and Bio-Plex Human ICAM-VCAM (hICAM-hVCAM) (Bio-Rad, Hercules, CA, USA) following the manufacturer’s protocol.

### 4.6. Statistics

The analyses were conducted using GraphPad Prism Version 9 (GraphPad Software, San Diego, CA, USA). Continuous variables were expressed as the mean ± standard deviation and categorical variables were expressed as number and percentage. The data were subjected to the Kolmogorov–Smirnov normality test and Bartlett’s test for homogeneity of variance. Log transformations were performed when appropriate. The categorical variables were analyzed using the Chi-squared test or Fisher’s exact test, and the continuous variables using unpaired Student’s *t*-test as appropriate. A log-rank test was applied to compare the ICU length of stay and ventilation duration. All *p*-values were two-sided, and *p* < 0.05 was considered statistically significant.

## 5. Conclusions

In conclusion, treatment of critical COVID-19 patients with low dose DXM resulted in a reduction of the inflammatory and lymphoid immune response but had little impact on the myeloid immune response, and no effect on endothelial activation, NETosis formation, and COVID-19-induced coagulopathy with persistent elevated levels of circulating TF.

## Figures and Tables

**Figure 1:**
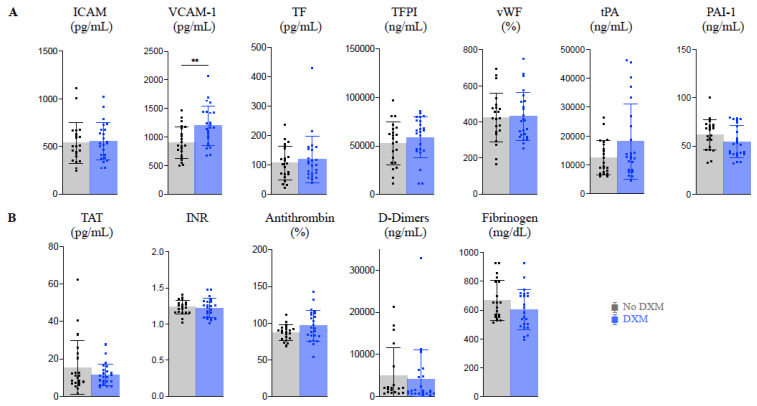
Corticosteroid effects on endothelial activation (**A**) and coagulation (**B**) in critical COVID-19 patients. Scatter graphs of soluble biomarkers of endothelial activation and coagulation. Individual values (dots), mean (colored rectangle), and standard deviation are presented on the graphs. ICAM, intercellular adhesion molecule; INR, international normalized ratio; PAI-1, plasminogen activator inhibitor-1; TAT, thrombin–antithrombin complex; TF, tissue factor; TFPI, tissue factor pathway inhibitor; tPA, tissue plasminogen activator; VCAM, vascular cell adhesion molecule; vWF, von Willebrand factor. ** *p* < 0.01.

**Figure 2:**
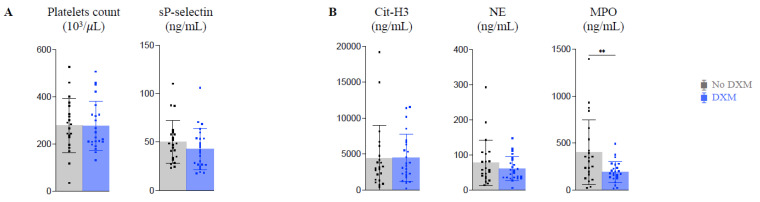
Corticosteroid effects on platelet activation (**A**) and NETosis (**B**) in critical COVID-19 patients. Scatter graphs of soluble biomarkers of platelet activation and NETosis. Individual values (dots), mean (colored rectangle), and standard deviation are presented on the graphs. ** *p* < 0.01 between the no DXM group and the DXM group. sP-selectin, soluble p-selectin; Cit-H3, citrullinated histone 3; MPO, myeloperoxidase; NE, neutrophil elastase.

**Table 1:** Baseline characteristics and clinical outcomes of patients treated or not with DXM.

	No DXM Group n = 22	DXM Group n = 24	*p* Value
DEMOGRAPHICS			
Men	15 (68)	19 (79)	0.51
Women	7 (32)	5 (21)	
Age (years)	59.9 ± 10.4	64.45 ± 8.9	0.12
MEDICAL HISTORY			
Hypertension	13 (59)	17 (71)	0.54
BMI > 25	15 (68)	17 (71)	>0.99
Diabetes	5 (23)	10 (42)	0.22
History of smoking	2 (9)	1(4)	0.60
COPD	3 (14)	1 (4)	0.34
CKD	0 (0)	2 (8)	0.49
Cancer	0 (0)	4 (17)	0.11
ROUTINE LABORATORY TESTING			
CRP (mg/dL)	199.3 ± 88.9	118.8 ± 76.6	<0.01 *
Highest CRP (mg/dL)	315.0 ± 126.4	285.8 ± 104.6	0.40
Creatinine (mg/dL)	0.92 ± 0.60	1.46 ± 2.44	0.40
Hemoglobin (g/dL)	11.2 ± 2.1	12.4 ± 2.2	0.07
WBCs (/µL)	9497 ± 3563	10,739 ± 4167	0.29
Neutrophils (/µL)	7803 ± 3170	9073 ± 3762	0.22
Lymphocytes (/µL)	894 ± 329	802 ± 425	0.27
Lowest lymphocyte count (/µL)	459 ± 323	504 ± 294	0.64
ORGAN FAILURE AND SEVERITY SCORES			
PaO_2_/FiO_2_	103 ± 4	86 ± 3	0.04 *
Apache II score	15 ± 4	14 ± 4	0.49
SOFA Score	4 ± 1	6 ± 2	<0.01 *
SIC score	0 (0)	0 (0)	>0.99
DIC score	0 (0)	0 (0)	>0.99
TREATMENT BEFORE INCLUSION			
Delay symptoms—inclusion (days)	10 ± 5	10 ± 4	0.87
Delay steroids—inclusion (days)	1.0	3.0 ± 2.6	NA
Hydroxychloroquine	19 (86)	0 (0)	<0.01
OUTCOME			
30-day mortality	6 (27)	9 (37)	0.54
1-year mortality	8 (36)	12 (50)	0.39
ICU length of stay (days)	27 ± 26	38 ± 42	<0.05 *
Ventilation duration (days)	21 ± 24	30 ± 35	0.08
Thrombo-embolic event	6 (27)	3 (13)	0.28

Values are numbers (percentages) or mean ± standard deviation. * means *p* value < 0.05. APACHE, acute physiology and chronic health evaluation; BMI, body mass index; COPD, chronic obstructive pulmonary disease; CKD, chronic kidney disease; CRP, C-reactive protein; DIC, disseminated intravascular coagulopathy; ICU, intensive care unit; PaO_2_/FiO_2_, arterial oxygen partial pressure/fractional inspired oxygen; SIC, sepsis-induced coagulopathy; SOFA, sepsis-related organ failure assessment; WBCs, white blood cells.

## Data Availability

The datasets analyzed during the current study are available from the corresponding author on reasonable request.

## Supplementary Materials

The supporting information can be downloaded at: https://www.mdpi.com/article/10.3390/ijms24087278/s1.
