# Supplementary material for: Dexamethasone Modulates the Cytokine Response but Not COVID-19-Induced Coagulopathy in Critically Ill
Source: Int J Mol Sci. 2023 Apr 14;24(8):7278. doi: 10.3390/ijms24087278 (PMC10138864; doi:10.3390/ijms24087278)
Supplement: Supplementary file 1 [file ijms-24-07278-s001.zip › ijms-2289269-supplementary.pdf]

Dexamethasone modulates the cytokine response but not COVID-19-induced coagulopathy in critically ill.

Dechamps et al, IJMS.

Supplementary Table S1: Reagents used for plasma biomarkers measurements.

Biomarker	Company	Catalog number
TF	R&D systems Minneapolis, Canada	#DCF300
TFPI	R&D systems Minneapolis, Canada	#DTFP10
vWF	HemosIL Lexington, USA	#0020002300
PAI-1	T coag Stago TriniLIZE, Marseille, France	#T6003
tPA	R&D systems Minneapolis, Canada	#DTPA00
ICAM-1	Bio-Rad, Hercules, California, USA	#171B6009M
VCAM-1	Bio-Rad, Hercules, California, USA	#171B6009M
TAT	R&D systems Minneapolis, Canada	#OWMG15
Antithrombin III	HemosIL Lexington, USA	#0020030100
sCD62P	R&D systems Minneapolis, Canada	#DPSE00
MPO	R&D systems Minneapolis, Canada	#DMYE00B
Cit-H3	Cayman, Michigan, USA	#501620
IFNγ	Bio-Rad, Hercules, California, USA	#M500KCAF0Y
IL-1β	Bio-Rad, Hercules, California, USA	#M500KCAF0Y
IL-1ra	Bio-Rad, Hercules, California, USA	#M500KCAF0Y
IL-2	Bio-Rad, Hercules, California, USA	#M500KCAF0Y
IL-4	Bio-Rad, Hercules, California, USA	#M500KCAF0Y
IL-6	Bio-Rad, Hercules, California, USA	#M500KCAF0Y
IL-7	Bio-Rad, Hercules, California, USA	#M500KCAF0Y
IL-8	Bio-Rad, Hercules, California, USA	#M500KCAF0Y
IL-10	Bio-Rad, Hercules, California, USA	#M500KCAF0Y
IL-12	Bio-Rad, Hercules, California, USA	#M500KCAF0Y
IL-13	Bio-Rad, Hercules, California, USA	#M500KCAF0Y
IL-17	Bio-Rad, Hercules, California, USA	#M500KCAF0Y
IP-10	Bio-Rad, Hercules, California, USA	#M500KCAF0Y
MCP-1	Bio-Rad, Hercules, California, USA	#M500KCAF0Y
MIP-1	Bio-Rad, Hercules, California, USA	#M500KCAF0Y
sCD40L	R&D systems Minneapolis, Canada	#DCDL40
sTREM-1	R&D systems Minneapolis, Canada	#DTRM10C

Abbreviation: TF, tissue factor; TFPI, tissue factor pathway inhibitor; vWF, von Willebrand factor; PAI-1, plasminogen activator inhibitor-1; tPA, tissue plasminogen activator; ICAM-1, intercellular adhesion molecule-1; TATc, thrombin antithrombin complex; ATIII, antithrombin III; MPO, myeloperoxidase; Cit-H3, citrullinated histone H3; IFNγ, interferon gamma; IL, interleukin; IL-1ra, IL-1 receptor antagonist; IP-10, IFNγ-induced protein 10; MCP-1, monocyte chemoattractant protein-1; MIP-1, macrophage inflammatory protein 1; sTREM-1, soluble triggering receptor expressed on myeloid cells 1.
